# Educational technologies for the prevention and management of extravasation of antineoplastic agents: an integrative review

**DOI:** 10.1590/0034-7167-2025-0308

**Published:** 2026-08-21

**Authors:** Rafael Fernando Mendes Barbosa, Larissa Bueno dos Santos, Josyane Borges da Silva Bonfim, Fabrine Aguilar Jardim, Moema Santos Souza, Namie Okino Sawada

**Affiliations:** I Universidade do Estado de Minas Gerais. Passos, Minas Gerais, Brazil; II Universidade Federal de Minas Gerais. Belo Horizonte, Minas Gerais, Brazil; III Universidade Federal de Alfenas. Alfenas, Minas Gerais, Brazil

**Keywords:** Nursing, Antineoplastic Agents, Extravasation of Diagnostic and Therapeutic Materials, Validation Study, Educational Technology., Enfermería, Antineoplásicos, Extravasación de Materiales Terapéuticos y Diagnósticos, Estudio de Validación, Tecnología Educacional.

## Abstract

**Objectives:**

to identify available scientific evidence on educational technologies for the prevention and management of extravasation of antineoplastic agents.

**Methods:**

an integrative review was conducted in November 2024, searching the MEDLINE (via PubMed), CINAHL (EBSCO), *Web of Science*, Scopus, Embase, BDENF, LILACS, and SciELO databases.

**Results:**

in total, 2,272 educational technologies were found, of which three comprised the final sample. The identified educational technologies presented variety in the design of pedagogical instruments focused on addressing the extravasation of antineoplastic substances, such as bundles, educational videos, and clinical protocols. In summary, they proved to be innovative strategies in the care of cancer patients, in addition to acting as didactic resources that enhance and facilitate access to evidence-based practice.

**Conclusions:**

educational technologies contribute to improving the quality of oncological care in clinical practice, proving to be effective strategies in training nurses and strengthening safer and more well-founded care.

## RESUMEN

**Objetivos:**

identificar la evidencia científica disponible sobre tecnologías educativas para la prevención y el manejo de la extravasación de agentes antineoplásicos.

**Métodos:**

se realizó una revisión integrativa en noviembre de 2024, con búsquedas en las bases de datos MEDLINE (vía PubMed), CINAHL (EBSCO), *Web of Science*, Scopus, Embase, BDENF, LILACS y SciELO.

**Resultados:**

se encontraron 2.272 resultados, de los cuales tres constituyeron la muestra final. Las tecnologías educativas identificadas presentaron una variedad en el diseño de instrumentos pedagógicos enfocados en abordar la extravasación de sustancias antineoplásicas, como bundles de ayuda, videos educativos y protocolos clínicos. En resumen, demostraron ser estrategias innovadoras en la atención a pacientes con cáncer, además de actuar como recursos didácticos que mejoran y facilitan el acceso a la práctica basada en la evidencia.

**Conclusiones:**

las tecnologías educativas contribuyen a mejorar la calidad de la atención oncológica en la práctica clínica, ya que demuestran ser estrategias efectivas en la formación de enfermeras y el fortalecimiento de una atención más segura e informada.

## INTRODUCTION

Extravasation of antineoplastic agents is characterized by the unintentional release of vesicant drugs from the vascular lumen into adjacent tissues. This complication can cause various clinical manifestations, including pain, swelling, redness, tissue damage, blistering, skin peeling, cell necrosis, and high morbidity. In more severe cases, surgical intervention may be necessary, with the possibility of significant functional sequels^(1,2)^.

The dispersion of these chemical agents represents a highly relevant clinical oncological emergency. Considering the specific mechanism of action of antineoplastic drugs, the consequences of extravasation, its associated complications, and the recommended therapeutic strategies are extensively explored in scientific studies^(3-5)^.

Research investigating the level of understanding among nursing staff regarding the extravasation of antineoplastic agents indicates that a significant portion of nurses working in oncology have difficulty accurately describing recommended management protocols or are unaware of the appropriate actions to be taken in the face of this adverse event. These findings highlight substantial gaps in the understanding of the essential care required in this context^(6-8)^.

The safe administration of chemotherapy is a responsibility that involves the entire multidisciplinary team^(7,8)^. However, nursing professionals play an essential role in providing this care, and are therefore more vulnerable to potential complications arising from extravasation. In this regard, it is crucial that these professionals not only understand the risks involved, but also possess the necessary technical skills, recognize the importance of strict adherence to protocols, and minimize distractions and interruptions during the administration of antineoplastic agents^(6-9)^.

Therefore, nurses working in oncology care must possess in-depth knowledge of adverse events associated with extravasation of antineoplastic agents, encompassing both preventive measures and appropriate management strategies^(3-5)^. This process can be promoted through educational practices, especially through the implementation of educational technologies, which enhance learning and facilitate the acquisition of knowledge and skills^(6-9)^.

Educational technology refers to processes based on experience, with the purpose of improving knowledge for its practical application in a specific context. Therefore, these processes aim to facilitate and improve educational practices, favoring the enhancement of teaching and learning processes for those involved^(10-14)^.

In the context of professional training aimed at caring for oncology patients undergoing chemotherapy, educational technologies have proven to be effective resources for enabling structured, efficient, and high-quality care^(13,14)^. Resources such as training, verbal instructions, informational materials, apps, videos, and educational games constitute innovative strategies in health education, benefiting both nursing professionals and oncology patients. Furthermore, these technologies broaden the reach of professional qualification, provide temporal and geographical flexibility, and reduce costs. Evidence indicates that these methodologies favor updating and technical improvement, positively impacting the implementation of interventions in clinical practice^(15,16)^.

Hence, the integration of educational technologies in the preventive approach and management of care in cases of extravasation is fundamental to underpinning clinical nursing practice, promoting comprehensive and qualified care for cancer patients. Its application contributes to optimizing care, improving overall health status, and increasing patients’ positive perception, with the added advantage of reducing costs, prolonged hospital care, and mitigating complications associated with the event. Thus, this review becomes relevant due to the demand to broaden the understanding of health and nursing professionals by identifying and making available educational technologies aimed at managing chemotherapy extravasation, contributing to the improvement of clinical practice.

## OBJECTIVES

To identify available scientific evidence on educational technologies for the prevention and management of extravasation of antineoplastic agents.

## METHODS

### Ethical aspects

This study strictly adhered to ethical standards, with all authors of the analyzed publications duly cited, ensuring the faithful presentation of the content, in accordance with Copyright Law 9,610/1998.

### Study design

This study used integrative review (IR) as a technique to consolidate knowledge. The stages performed included formulating the research question, selecting the sample, categorizing the studies, assessing the included articles, interpreting the data, and synthesizing the findings^(17)^.

IR registration was made on Open Science Framework, and the protocol is available at the following link: https://osf.io/xp9vh/, having the DOI identifier: 10.17605/OSF.IO/XP9VH^(18)^.

### Research question formulation

To formulate the question, the acronym PCC (Population, Concept, and Context) was adopted, where P = people with cancer, C = prevention and management of extravasation of antineoplastic agents, and C = educational technologies. Thus, the guiding question of this study was: what educational technologies exist for the prevention and management of extravasation of antineoplastic agents in people with cancer?

### Literature search and sampling

Data collection involved searching eight information databases, namely: Medical Literature Analysis and Retrieval System online (via PubMed^®^); Cumulative Index to Nursing and Allied Health Literature (CINAHL-Ebsco); Web of Science; Scopus; Embase; Nursing Database; Latin American and Caribbean Literature in Health Sciences, accessed through the Virtual Health Library; and Scientific Electronic Library Online.

The research was conducted using the Coordination for the Improvement of Higher Education Personnel Journal Portal, accessed via the *Comunidade Acadêmica Federada* of the *Universidade do Estado de Minas Gerais*. The search terms were selected from Medical Subject Headings, CINAHL Headings, Health Sciences Descriptors, and Embase thesaurus, used for Embase. The search strategies were applied to the databases during November 2024, as shown in Chart 1.

**Chart 1:** Search procedures implemented in specific information databases, 2025

Database	Search strategy
MEDLINE	((“Nursing Care”[Mesh] OR “Patient Care Bundles”[Mesh] OR “Accident Prevention”[Mesh]) AND (“Extravasation of Diagnostic and Therapeutic Materials”[Mesh] OR “Antineoplastic Agents”[Mesh] OR “Drug Therapy” OR “Chemotherapy, Adjuvant”[Mesh] OR “Induction Chemotherapy”[Mesh] OR “Consolidation Chemotherapy” [Mesh] OR “Maintenance Chemotherapy”[Mesh] OR “Medication Therapy Management”[Mesh] OR “Administration and Dosage” OR “Antineoplastic Combined Chemotherapy Protocols”[Mesh] OR “Injection Site Reaction”[Mesh])) AND (“Educational Technology”[Mesh] OR “Multimedia”[Mesh] OR “Teaching Materials”[Mesh] OR “Instructional Film and Video” OR “Communications Media”[Mesh] OR “Health Education”[Mesh] OR “Validation Study” OR “Validation Studies”)
Embase	(‘nursing care’/exp OR ‘care bundle’/exp OR ‘accident prevention’/exp) AND (‘contrast medium extravasation’/exp OR ‘antineoplastic agent’/exp OR ‘antineoplastic agents’ OR ‘adjuvant chemotherapy’/exp OR ‘induction chemotherapy’/exp OR ‘consolidation chemotherapy’/exp OR ‘maintenance chemotherapy’/exp OR ‘medication therapy management’/exp OR ‘administration and dosage’/exp OR ‘injection site reaction’/exp) AND (‘educational technology’/exp OR ‘multimedia’/exp OR ‘teaching’/exp OR ‘video assisted technique’/exp OR ‘mass medium’/exp OR ‘health education’/exp OR ‘validation studies’ OR ‘validation studies as topic’ OR ‘validation study’/exp)
Web of Science	ALL=(“Nursing Care” OR “Patient Care Bundles” OR “Accident Prevention” ) AND ALL=(“Extravasation of Diagnostic and Therapeutic Materials” OR “Antineoplastic Agents” OR “Drug Therapy” OR “Chemotherapy, Adjuvant” OR “Induction Chemotherapy” OR “Consolidation Chemotherapy” OR “Maintenance Chemotherapy” OR “Medication Therapy Management” OR “Administration and Dosage” OR “Antineoplastic Combined Chemotherapy Protocols” OR “Injection Site Reaction”) AND ALL=(“Educational Technology” OR “Multimedia” OR “Teaching Materials” OR “Instructional Film and Video” OR “Communications Media” OR “Health Education” OR “Validation Study” OR “Validation Studies”)
Scopus	(( ALL ( “Nursing Care” OR “Patient Care Bundles” OR “Accident Prevention” ) ) AND ( ALL ( “Extravasation of Diagnostic and Therapeutic Materials” OR “Antineoplastic Agents” OR “Drug Therapy” OR “Chemotherapy, Adjuvant” OR “Induction Chemotherapy” OR “Consolidation Chemotherapy” OR “Maintenance Chemotherapy” OR “Medication Therapy Management” OR “Administration and Dosage” OR “Antineoplastic Combined Chemotherapy Protocols” OR “Injection Site Reaction” ) ) AND ( ALL ( “Educational Technology” OR “Multimedia” OR “Teaching Materials” OR “Instructional Film and Video” OR “Communications Media” OR “Health Education” OR “Validation Study” OR “Validation Studies” ) )
CINAHL	( MH “Nursing Care” OR “Nursing Care” OR “Patient Care Bundles” OR MH “Safety” OR “Accident Prevention” ) AND ( MH “Extravasation of Diagnostic and Therapeutic Materials” OR “Extravasation of Diagnostic and Therapeutic Materials” OR MH “Antineoplastic Agents” OR “Antineoplastic Agents” OR MH “Drug Therapy” OR “Drug Therapy” OR MH “Chemotherapy, Adjuvant” OR “Chemotherapy, Adjuvant” OR MH “Induction Chemotherapy” OR “Induction Chemotherapy” OR MH “Consolidation Chemotherapy” OR “Consolidation Chemotherapy” OR “Maintenance Chemotherapy” OR MH “Medication Management” OR “Medication Therapy Management” OR “Administration and Dosage” OR MH “Antineoplastic Agents, Combined” OR “Antineoplastic Combined Chemotherapy Protocols” OR MH “Injection Sites” OR “Injection Site Reaction” ) AND ( MH “Educational Technology” OR “Educational Technology” OR MH “Multimedia” OR “Multimedia” OR MH “Teaching Materials” OR “Teaching Materials” OR “Instructional Film and Video” OR MH “Communications Media” OR “Communications Media” OR MH “Health Education” OR “Health Education” OR MH “Validation Studies” OR “Validation Study”)
SciELO	((Nursing Care) OR (Patient Care Bundles) OR (Accident Prevention) ) AND ((Extravasation of Diagnostic and Therapeutic Materials) OR (Antineoplastic Agents) OR (Drug Therapy) OR (Chemotherapy, Adjuvant) OR (Induction Chemotherapy) OR (Consolidation Chemotherapy) OR (Maintenance Chemotherapy) OR (Medication Therapy Management) OR (Administration and Dosage) OR (Antineoplastic Combined Chemotherapy Protocols) OR (Injection Site Reaction)) AND ((Educational Technology) OR (Multimedia) OR (Teaching Materials) OR (Instructional Film and Video) OR (Communications Media) OR (Health Education) OR (Validation Study) OR (Validation Studies))
LILACS	((Nursing Care) OR (Patient Care Bundles) OR (Accident Prevention)) AND ((Extravasation of Diagnostic and Therapeutic Materials) OR (Antineoplastic Agents) OR (Drug Therapy) OR (Chemotherapy, Adjuvant) OR (Induction Chemotherapy) OR (Consolidation Chemotherapy) OR (Maintenance Chemotherapy) OR (Medication Therapy Management) OR (Administration and Dosage) OR (Antineoplastic Combined Chemotherapy Protocols) OR (Injection Site Reaction)) AND ((Educational Technology) OR (Multimedia) OR (Teaching Materials) OR (Instructional Film and Video) OR (Communications Media) OR (Health Education) OR (Validation Study) OR (Validation Studies))
BDENF	((Nursing Care) OR (Patient Care Bundles) OR (Accident Prevention)) AND ((Extravasation of Diagnostic and Therapeutic Materials) OR (Antineoplastic Agents) OR (Drug Therapy) OR (Chemotherapy, Adjuvant) OR (Induction Chemotherapy) OR (Consolidation Chemotherapy) OR (Maintenance Chemotherapy) OR (Medication Therapy Management) OR (Administration and Dosage) OR (Antineoplastic Combined Chemotherapy Protocols) OR (Injection Site Reaction)) AND ((Educational Technology) OR (Multimedia) OR (Teaching Materials) OR (Instructional Film and Video) OR (Communications Media) OR (Health Education) OR (Validation Study) OR (Validation Studies))

Selection included primary studies regardless of language or year of publication. On the other hand, case studies, monographs, theses, dissertations, letters to the editor, abstracts, opinion articles, correspondence, reviews, and book chapters were not considered.

After applying the search strategy to each database, the results were imported into the EndNote X7 reference management software (Desktop version), where duplicate articles were eliminated^(17)^. Study selection was conducted using the free Rayyan platform, developed by the Qatar Computing Research Institute, in order to identify those that met the previously defined inclusion criteria^(19)^.

The preliminary selection stage consisted of assessing the papers’ titles and abstracts to verify their compliance with IR eligibility criteria. Subsequently, the approved articles underwent a full reading. Both stages were conducted independently and blindly by two reviewers, based on the research question and the established inclusion and exclusion criteria. In case of disagreement among the reviewers, a third expert in the field was consulted to resolve the differences.

In addition to the database searches, the reviewers also conducted a manual survey of the bibliographic references of the primary studies included in the IR.

### Study characterization and assessment

Data collection was carried out using a revised instrument that included information such as reference and year of publication, study objective, methodological characteristics (including the study design as described by the authors and the sample size), and the main findings related to educational technologies applied in the prevention and management of extravasation of antineoplastic agents^(20)^.

After this stage, data interpretation was carried out qualitatively, through the descriptive synthesis of evidence from primary studies. As described by the authors of the works incorporated in the IR. For the critical appraisal phase, the Johns Hopkins Nursing Evidence-Based Practice tool^(21)^ was chosen to assess the methodological consistency of the selected works. This assessment was conducted independently by two reviewers, who applied the specific instrument for the corresponding study design - whether quantitative, qualitative or mixed -, which uses responses categorized as “yes”, “no” or “not applicable”. In the stage prior to critical assessment, the reviewers jointly defined the criteria for judgment and, in situations of disagreement, the decision was left to a third reviewer who was called upon to mediate and resolve the conflicts that arose.

The selected studies were classified according to methodological quality, divided into three categories: high, good, and low quality. High-quality studies presented solid and applicable results, samples adequate to the research design, rigorous controls, conclusive conclusions, and recommendations based on a broad literature review, including complete references to scientific evidence. Those considered of good quality showed relatively consistent results, sufficient samples for study design, some level of control, justified conclusions, and recommendations based on comprehensive reviews, albeit with partial references to evidence. Finally, low-quality studies demonstrated limited evidence and inconsistent results, samples inadequate to the design, and inconclusive conclusions^(21)^.

Recognizing the importance of combining methodological quality with the level of evidence to support decisions in clinical practice, the selected studies were organized according to the levels of evidence established by the Johns Hopkins Nursing Evidence-Based Practice hierarchical model^(21)^.

## RESULTS

**Figure 1:**
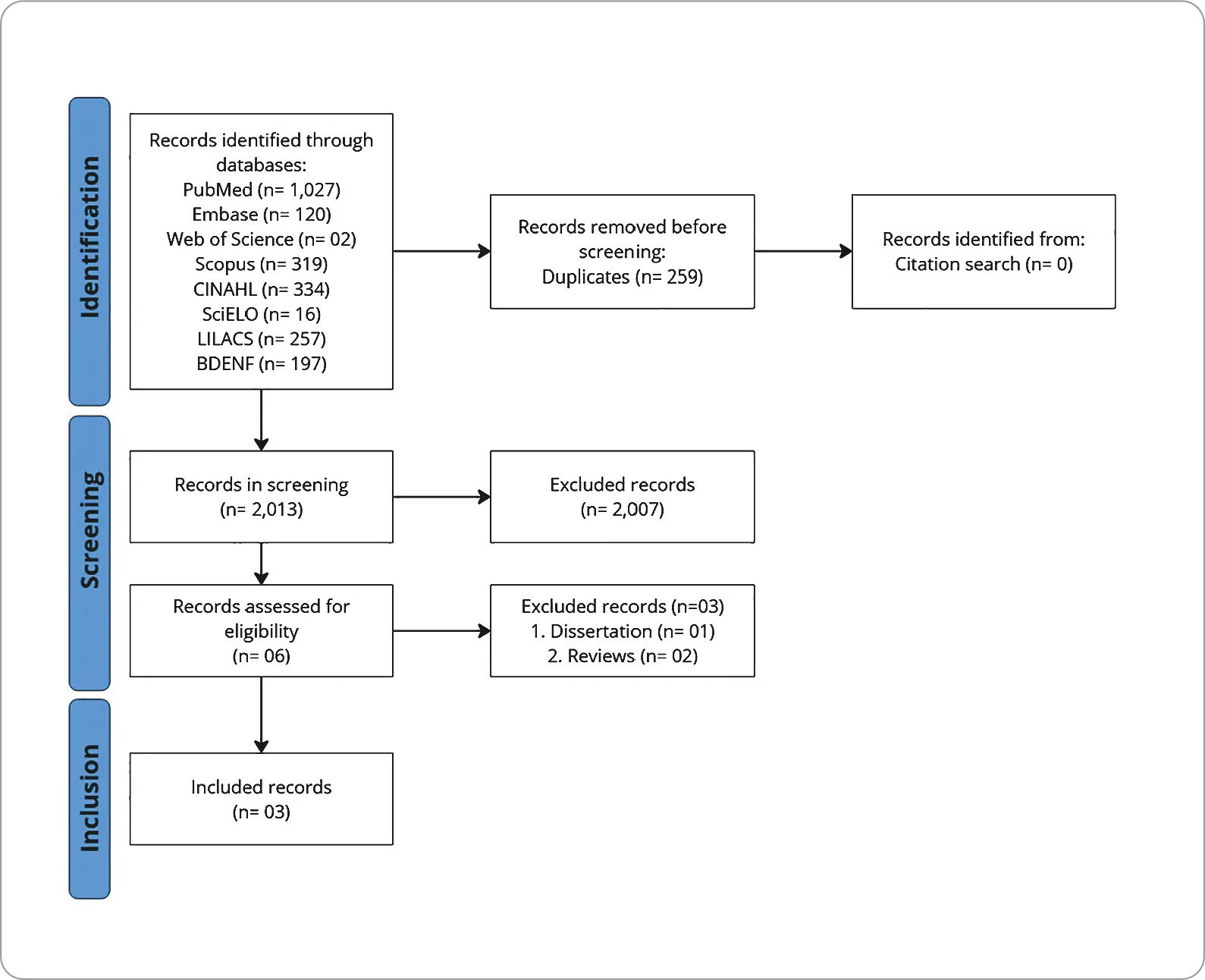
Graphical representation of the flow of identification, screening, and inclusion of studies in the integrative review, 2025

The database search yielded a total of 2,272 retrieved records, of which 259 were identified as duplicates and excluded. After initial screening based on titles and abstracts, according to established inclusion and exclusion criteria, six articles were selected for full-text reading. In the next stage, a complete analysis of the texts resulted in the inclusion of three publications in this journal. Figure 1 shows the detailed flow of study identification and selection.

Chart 2 presents a descriptive overview of the primary studies, with information on authors, place and year of publication, study design, sample and objective, as well as main results and conclusions, type and classification of educational technology, methodological quality and level of evidence, limitations of each study, context of application, and practical impact.

**Chart 2:** Characterization of the studies included in the integrative review, 2025

Authors/location/year	Study design/sample	Objective	Main results/conclusions	Type/classification of educational technology/Johns Hopkins/NE	Study limitations	Application context	Practical impact
Melo *et al*., 2020, Brazil^(22)^	Methodological study, developed in three phases: conducting a scoping review, constructing the bundle, and content assessment by experts. The sample consisted of 13 judges in the first Delphi stage and nine judges in the second Delphi stage. All were specialist nurses in oncology.	Construct and assess the content of a bundle (set of measures) for prevention and management of extravasation of antineoplastic agents in adult cancer patients.	The study validated a management bundle for extravasation of vesicant drugs, showing that it possesses acceptable psychometric properties and high reliability, with recommendations for changes implemented by judges. The assessment used the Delphi technique and achieved high agreement and significance. The bundle obtained a Content Validity Coefficient of 0.93 for prevention and 0.96 for management, highlighting the importance of early detection to prevent greater harm. The implementation of the bundle can improve the quality of oncological care, requiring professional training and periodic assessments to ensure its efficiency and applicability.	Bundle/soft-hard/high quality/IIIc	Low number of responses from experts.	Large hospital with a High Complexity Oncology Unit.	Strengthen nurses’ attention regarding the management of procedures related to extravasation of vesicant drugs in cancer patients.
Freitas, *et al*., 2022, Brazil^(23)^	This is a descriptive study of a qualitative nature. The sample consisted of a group of specialist nurses.	Report on the experience in developing and implementing the protocol for extravasation and infiltration of antineoplastic agents via central venous access.	The implementation of a protocol for administering antineoplastic agents significantly reduced complications, standardizing procedures and increasing the confidence of healthcare professionals. The protocol facilitated the early identification of signs of infiltration and extravasation, allowing for rapid interventions. Standardization improved the safety and efficiency of clinical practices. A care flowchart was implemented in an oncology outpatient clinic in May 2021, accompanied by staff training.	Protocol/soft-hard/low quality/IIIc	The study does not explicitly state its limitations.	Oncology outpatient clinic of the *Hospital Estadual de Botucatu*, affiliated with the *Hospital das Clínicas da Faculdade de Medicina de Botucatu*.	Acquisition of knowledge and technical skills for managing the infiltration and extravasation of antineoplastic agents in central venous access.
Barbosa *et al*., 2024, Brazil^(9)^	This methodological study, developed according to Falkembach’s theoretical framework, which advocates five stages in the construction of educational video media: analysis and planning, modeling, implementation, assessment, and distribution. The sample consisted of nine judges specializing in oncology and five judges specializing in Technology and Information.	Create and develop an educational video about the prevention and management of extravasation of antineoplastic agents aimed at nursing professionals.	The 18-minute educational video on managing chemotherapy extravasation achieved a Content Validity Index of 90.8%, with 94.2% between the assessments of content and technique judges. Combining narratives and simulated scenes, the video addresses risk factors, preventive actions, and proper management, with subtitles to facilitate understanding. Well-received by the target audience and judges, it was considered an effective method for training nurses and promoting evidence-based nursing practices.	Educational video/soft-hard/high quality/IIIc	The script and storyboard size in terms of content; the final video length; the absence of subtitles in other languages; and the lack of resources for teaching sign language to people with hearing impairments.	Nursing professionals working in outpatient chemotherapy services, specifically in the administration of antineoplastic agents.	The topic was addressed in an accessible way on open-access digital platforms, with the potential to improve care for people with cancer, overcoming geographical limitations.

Based on the results, it was possible to identify that educational technologies presented diverse approaches in the development of didactic resources for the management of chemotherapy extravasation, such as bundles, educational videos, and clinical protocols. These technologies were classified as soft-hard, as they articulate structured knowledge with elements of healthcare work.

The methodological approach to the implementation process, as well as the use of a methodological foundation for the development of educational technologies included in this review, were described in only two studies, demonstrating that the methodological process phases were correctly implemented and/or detailed by the authors, including the specification of the method used to create teaching resources, ensuring rigor in its execution and proving to be reliable.

In summary, despite their limitations, the technologies have demonstrated that their impact on clinical practice represents innovative strategies in the care provided within their application context, in addition to building didactic resources that enhance and facilitate access to evidence-based practice directed at nursing professionals. It is noteworthy that, in all the studies analyzed, prevention and management actions were conducted by nurses specialized in oncology.

## DISCUSSION

The development of educational technologies to guide professionals in dealing with chemotherapy extravasation remains a significant gap in clinical nursing practice. Extravasation of antineoplastic agents can cause severe complications, such as tissue necrosis, intense pain, and functional loss of affected limbs^(1,2)^. Given the complexity of cancer treatment and the significant risk of harm to patients, it is essential that the nursing team maintain constant updates on the adoption of safe practices to prevent and treat these events^(9,22,23)^.

In this context, institutions responsible for health training and assistance need to invest in the development of educational technologies as an educational resource, in order to perpetuate innovative training cycles through a constructivist and collaborative approach, and to enable educational tools that bring about changes in the implementation of effective strategies for nursing care^(24)^.

The included studies^(9,22,23)^ gathered evidence about educational technologies to support the prevention and management of spill-related incidents, demonstrating that there are few studies focused on the development and implementation of specific educational tools on the subject, in addition to identifying that the methodological approach that ensures rigor in its elaboration was employed in only two studies^(9,22)^, ensuring the reliability of the technology developed.

In a field like oncology, where new drugs and protocols are frequently introduced, the constant updating of knowledge among nursing professionals is indispensable^(6-8)^. Without access to effective educational tools, professionals may not be adequately prepared to identify early signs of leakage or to implement necessary interventions efficiently, potentially resulting in inadequate or delayed responses and increasing the risk of serious complications for patients^(25)^.

Technologies have the potential to create collaborative platforms that allow for the widespread dissemination of knowledge and experiences among healthcare professionals. However, without studies and initiatives that promote these tools, opportunities for collective learning are limited, reducing the healthcare system’s ability to respond proactively and effectively to clinical challenges^(26)^. The adoption of technological educational tools in the training of nursing professionals has proven essential for the proper management of extravasation of antineoplastic agents, an oncological emergency that can cause serious harm^(9,22,23)^.

The synthesis presented here demonstrates that the integration of innovative educational tools, such as protocols^(23)^, videos^(9)^, and bundles^(22)^, offers crucial support for the continuous training and evidence-based practice of nurses involved in oncological care. The implementation of a protocol for the administration of antineoplastic drugs significantly reduces complications, standardizing procedures and increasing healthcare professionals’ confidence^(23)^. The development of educational resources in audiovisual format, for instance, is a powerful strategy in the context of oncology nursing and demonstrates that educational videos increase professionals’ understanding of extravasation prevention and management, promoting safe and evidence-based practices^(9)^. Furthermore, educational bundles, which consist of packages of evidence-based practices, have proven effective in standardizing care^(22)^.

Although the benefits mentioned are evident, each technology has strengths and limitations that must be assessed in the context of clinical practice. Educational videos, while accessible and low-cost, depend on the active engagement of the professional and do not replace practical supervision or in-person interaction. The clinical protocol, being less attractive from a pedagogical point of view, requires constant updating to remain aligned with the evidence. The bundle, on the other hand, represents a more robust strategy, as it brings together integrated interventions with potential impact on clinical outcomes; however, its implementation demands more institutional effort, including training, continuous monitoring, and resource availability^(27-29)^. Therefore, the choice between these technologies, or a combination of them, should consider not only their isolated effectiveness, but also organizational contexts, the prevailing safety culture, and the resources available in healthcare services.

Additionally, the application of these educational technologies must be aligned with international guidelines for cancer patient safety. Organizations such as the Oncology Nursing Society and the Centers for Disease Control and Prevention emphasize the importance of standardized practices, effective communication, and continuing education for healthcare staff to prevent complications^(30,31)^. In this context, protocols and bundles offer support for the standardization and implementation of evidence-based measures, while educational videos contribute to the understanding and adherence to recommended practices^(9,22,23)^. In this way, the integration of these technologies not only strengthens the continuing training of nurses, but also promotes compliance with international recommendations, reinforcing safe and consistent practices in cancer care^(30,31)^.

Despite the demonstrated potential, the lack of widely available and validated educational technologies in the context of extravasation of antineoplastic agents represents a critical gap in oncology, with direct impacts on improving patient care and protection. The absence of these tools can result in ineffective management and increase healthcare professionals’ workload. Inadequate training can lead to the application of outdated and inconsistent techniques, as well as hinder the implementation of evidence-based practices^(6-8,32)^.

Educational technologies consist of knowledge expanded through human intervention, and not merely the design and implementation of tools. They constitute an ordered body of knowledge with a pedagogical purpose, based on scientific evidence, that enables the organization, implementation, mastery, and continuous assessment of the educational process^(10,33)^. From this perspective, the specificities of the teaching resources highlight the predominance of soft-hard technologies that contributed to the adoption of extravasation prevention and management measures, directed at nursing professionals, among the studies included in this review.

It is well known that technologies contribute to the nursing landscape, being applied for both care and educational purposes, recognized as an educational tool that involves multiple aspects, including text, sound, and images, in a single material for advancing knowledge^(34,35)^. In addition, its application enhances the skills of professionals in clinical practice, provides opportunities to acquire essential care skills, as it promotes learning and improves both team performance and satisfaction^(36,37)^.

Most studies presented level IIIc evidence, with two of them demonstrating high quality in methodological assessment, in addition to the use of a technical foundation to guide the phases of developing the educational resource. Finally, it is noteworthy that the development and use of educational technologies is increasingly evident in the training of nursing professionals, and aligned with this phenomenon, Brazilian nurses have shown a prominent position in the conception of this type of material.

Investing in educational technologies and research in this area is essential to improve quality of care and ensure that professionals are able to overcome the challenges associated with managing chemotherapy extravasation. It is urgent to address this gap by promoting an environment where continuing education and access to evidence-based information are guaranteed for all professionals involved in cancer care.

### Study limitations

A limitation of this study was the low number of publications identified on educational technologies related to the investigated topic, with only three studies found. This result may compromise the soundness of the conclusions, as the scarcity of records suggests a possible gap in the scientific production of the area. Furthermore, the reduced number of studies may reflect less concern or involvement of nursing professionals in the development of new technologies, highlighting the need for greater investment in the creation and dissemination of educational strategies. Another limitation is the studies’ geographical restriction, since all were developed in Brazil. This limitation prevents a broader view of the topic in different contexts and realities, hindering comparison with international experiences. This scenario reinforces the importance of new research in other regions and countries in order to expand knowledge about the development and application of educational technologies in the investigated area.

### Contributions to nursing

This educational journal makes significant contributions to the advancement of knowledge and practices for nursing professionals. Given the severity of complications associated with extravasation of antineoplastic agents, it is essential to develop educational materials that can fill these gaps. Well-founded studies can offer clear guidelines and evidence-based practices, contributing to standardization of care and minimization of risks for patients. Furthermore, by addressing current limitations, such studies encourage more comprehensive and diverse future research, promoting continuous improvement in the safety and efficacy of cancer treatment.

## CONCLUSIONS

The evidence consolidated in this review indicates that educational technologies for managing chemotherapy extravasation are still underexplored in the scientific literature, which may reflect a gap in the qualification of nursing professionals and in the adoption of systematized strategies for the safety of cancer patients.

However, considering the synthesized evidence, it is concluded that didactic resources contribute to the improvement of oncological care in clinical practice, constituting effective methods to train nurses, promote evidence-based practices in nursing, and reduce complications associated with extravasation. These strategies not only reinforce patient safety but also improve the quality of care provided.

## Data Availability

The research data are available within the article.
