# Supplementary material for: Educational technologies for the prevention and management of extravasation of antineoplastic agents: an integrative review
Source: Rev Bras Enferm. 2026 Aug 21;79:e20250308. doi: 10.1590/0034-7167-2025-0308 (PMC13505564; doi:10.1590/0034-7167-2025-0308)
Supplement: Translation

# Translation: Tecnologias educativas para prevenção e manejo do extravasamento de agentes antineoplásicos: revisão integrativa

**DOI:** 10.1590/0034-7167-2025-0308pt

**Authors:** Rafael Fernando Mendes Barbosa, Larissa Bueno dos Santos, Josyane Borges da Silva Bonfim, Fabrine Aguilar Jardim, Moema Santos Souza, Namie Okino Sawada

**Affiliations:** I Universidade do Estado de Minas Gerais. Passos, Minas Gerais, Brasil; II Universidade Federal de Minas Gerais. Belo Horizonte, Minas Gerais, Brasil; III Universidade Federal de Alfenas. Alfenas, Minas Gerais, Brasil

**Keywords:** Enfermagem, Antineoplásicos, Extravasamento de Materiais Terapêuticos e Diagnósticos, Estudos de Validação, Tecnologia Educacional.

## Abstract

**Objetivos:**

identificar as evidências científicas disponíveis sobre tecnologias educativas para prevenção e manejo do extravasamento de agentes antineoplásicos.

**Métodos:**

conduziu-se revisão integrativa em novembro de 2024, com busca nas bases de dados MEDLINE (via PubMed), CINAHL (EBSCO), *Web of Science*, Scopus, Embase, BDENF, LILACS e SciELO.

**Resultados:**

ao todo, 2.272 foram encontrados, dos quais três compuseram a amostra final. As tecnologias educativas identificadas apresentaram variedade na concepção de instrumentos pedagógicos com foco na abordagem do extravasamento de substâncias antineoplásicas, tais como *bundle*, vídeo educativo e protocolo clínico. Em síntese, revelaram-se estratégias inovadoras no cuidado de pacientes oncológicos, além de atuarem como recursos didáticos que potencializam e facilitam o acesso à prática baseada em evidências.

**Conclusões:**

as tecnologias educativas contribuem para qualificar o cuidado oncológico na prática clínica, uma vez que se mostram estratégias eficazes na capacitação de enfermeiros e no fortalecimento de uma assistência mais segura e fundamentada.

## INTRODUÇÃO

O extravasamento de agentes antineoplásicos é caracterizado pela liberação não intencional de drogas vesicantes do lúmen vascular para os tecidos adjacentes. Essa complicação pode provocar diferentes manifestações clínicas, incluindo dor, inchaço, vermelhidão, lesões nos tecidos, formação de bolhas, descamação da pele, necrose celular e alta morbidade. Em situações mais severas, pode ser necessária intervenção cirúrgica, com possibilidade de sequelas funcionais relevantes^(1,2)^.

A dispersão desses agentes químicos representa uma emergência oncológica de elevada relevância clínica. Considerando o mecanismo de ação específico dos fármacos antineoplásicos, as consequências do extravasamento, suas complicações associadas e as estratégias terapêuticas recomendadas são amplamente exploradas em estudos científicos^(3-5)^.

Pesquisas que investigam o nível de compreensão da equipe de enfermagem acerca do extravasamento de agentes antineoplásicos indicam que uma parcela significativa dos enfermeiros que atuam na área oncológica apresenta dificuldades na descrição precisa dos protocolos de manejo recomendados ou desconhece as condutas apropriadas a serem adotadas diante desse evento adverso. Esses achados evidenciam lacunas substanciais no entendimento dos cuidados essenciais requeridos nesse contexto^(6-8)^.

A administração segura da quimioterapia é uma responsabilidade que envolve toda a equipe multiprofissional^(7,8)^. No entanto, os profissionais de enfermagem assumem uma função essencial na realização desse cuidado, estando, portanto, mais vulneráveis às possíveis complicações decorrentes do extravasamento. Nesse sentido, é fundamental que esses profissionais não apenas compreendam os riscos envolvidos, mas também possuam as competências técnicas necessárias, reconheçam a importância da adesão rigorosa aos protocolos e minimizem distrações e interrupções durante a administração dos agentes antineoplásicos^(6-9)^.

Assim, enfermeiros que atuam na assistência oncológica devem possuir um conhecimento aprofundado sobre os eventos adversos associados ao extravasamento de agentes antineoplásicos, abrangendo tanto medidas preventivas quanto estratégias de manejo adequadas^(3-5)^. Esse processo pode ser promovido por meio de práticas educativas, especialmente através da implementação de tecnologias educacionais, que potencializam a aprendizagem e facilitam a aquisição de conhecimentos e habilidades^(6-9)^.

A tecnologia educacional se refere a processos fundamentados em experiências, com o propósito de aprimorar conhecimentos para sua aplicação prática em um contexto específico. Dessa forma, esses processos têm como objetivo facilitar e aprimorar as práticas educativas, favorecendo o aprimoramento dos processos de ensino e aprendizado dos envolvidos^(10-14)^.

No âmbito da formação profissional direcionada ao atendimento de pacientes oncológicos em quimioterapia, as tecnologias educacionais têm se mostrado recursos eficazes para viabilizar um cuidado estruturado, eficiente e de alta qualidade^(13,14)^. Recursos como treinamentos, instruções verbais, materiais informativos, aplicativos, vídeos e jogos educativos configuram estratégias inovadoras na educação em saúde, beneficiando tanto os profissionais de enfermagem quanto os pacientes oncológicos. Além disso, tais tecnologias ampliam o alcance da qualificação profissional e proporcionam flexibilidade temporal e geográfica, além de custos reduzidos. Evidências indicam que essas metodologias favorecem a atualização e o aprimoramento técnico, impactando positivamente a implementação de intervenções na prática assistencial^(15,16)^.

Nesse sentido, a integração de tecnologias educativas na abordagem preventiva e na gestão de cuidados em casos de extravasamento é fundamental para embasar a prática clínica de enfermagem, promovendo uma assistência integral e qualificada ao paciente oncológico. Sua aplicação contribui para a otimização do cuidado, melhoria do estado geral de saúde e percepção positiva dos pacientes, com vantagem adicional de reduzir custos, cuidados hospitalares prolongados e mitigar complicações associadas ao evento. Assim, esta revisão se torna relevante em razão da demanda por ampliar o entendimento dos profissionais de saúde e enfermagem, ao identificar e disponibilizar tecnologias educativas voltadas ao gerenciamento do extravasamento de quimioterápicos, contribuindo para a qualificação da prática clínica.

## OBJETIVOS

Identificar as evidências científicas disponíveis sobre tecnologias educativas para prevenção e manejo do extravasamento de agentes antineoplásicos.

## MÉTODOS

### Aspectos éticos

Houve cumprimento rigoroso das normas éticas neste estudo com todos os autores das publicações analisadas devidamente citados, garantindo a apresentação fiel do conteúdo, em conformidade com a Lei de Direitos Autorais nº 9.610/1998.

### Tipo de estudo

Este estudo utilizou a revisão integrativa (RI) como técnica para consolidar o conhecimento. As etapas realizadas contemplaram a formulação da questão de pesquisa, a seleção da amostra, a categorização dos estudos, a avaliação dos artigos incluídos, a interpretação dos dados e a síntese dos achados^(17)^.

O registro da RI foi feito na plataforma *Open Science Framework*, estando o protocolo disponível no *link:* https://osf.io/xp9vh/, tendo o identificador DOI: 10.17605/OSF.IO/XP9VH^(18)^.

### Elaboração da questão de pesquisa

Para a elaboração da pergunta, adotou-se o acrônimo PCC (População, Conceito e Contexto), sendo P = pessoas com câncer, C = prevenção e manejo do extravasamento de agentes antineoplásicos e C= tecnologias educativas. Desta forma, a questão norteadora deste estudo foi definida como: quais são as tecnologias educativas existentes para a prevenção e manejo do extravasamento de agentes antineoplásicos em pessoas com câncer?

### Busca na literatura e amostragem

O levantamento de dados envolveu a busca em oito bases de informação, a saber: *Medical Literature Analysis and Retrieval System on-line* (MEDLINE via PubMed^®^); *Cumulative Index to Nursing and Allied Health Literature* (CINAHL-Ebsco); *Web of Science*; Scopus; Embase; Base de Dados em Enfermagem; Literatura Latino-Americana e do Caribe em Ciências da Saúde, acessada pela Biblioteca Virtual em Saúde; e *Scientific Electronic Library Online*.

A pesquisa foi conduzida por meio do Portal de Periódicos da Coordenação de Aperfeiçoamento de Pessoal de Nível Superior, com acesso realizado via Comunidade Acadêmica Federada da Universidade do Estado de Minas Gerais. Os termos de busca foram selecionados a partir dos descritores do *Medical Subject Headings*, CINAHL *Headings*, Descritores em Ciências da Saúde e*thesaurus* do Embase, utilizados para Embase. As estratégias de pesquisa foram aplicadas nas bases de dados durante o mês de novembro de 2024, conforme apresentado no Quadro 1.

**Quadro 1:** Procedimentos de busca implementados nas bases de informação específicas, 2025

Base de dados	Estratégia de busca
MEDLINE	*((“Nursing Care”[Mesh] OR “Patient Care Bundles”[Mesh] OR “Accident Prevention”[Mesh]) AND (“Extravasation of Diagnostic and Therapeutic Materials”[Mesh] OR “Antineoplastic Agents”[Mesh] OR “Drug Therapy” OR “Chemotherapy, Adjuvant”[Mesh] OR “Induction Chemotherapy”[Mesh] OR “Consolidation Chemotherapy” [Mesh] OR “Maintenance Chemotherapy”[Mesh] OR “Medication Therapy Management”[Mesh] OR “Administration and Dosage” OR “Antineoplastic Combined Chemotherapy Protocols”[Mesh] OR “Injection Site Reaction”[Mesh])) AND (“Educational Technology”[Mesh] OR “Multimedia”[Mesh] OR “Teaching Materials”[Mesh] OR “Instructional Film and Video” OR “Communications Media”[Mesh] OR “Health Education”[Mesh] OR “Validation Study” OR “Validation Studies”)*
Embase	*(‘nursing care’/exp OR ‘care bundle’/exp OR ‘accident prevention’/exp) AND (‘contrast medium extravasation’/exp OR ‘antineoplastic agent’/exp OR ‘antineoplastic agents’ OR ‘adjuvant chemotherapy’/exp OR ‘induction chemotherapy’/exp OR ‘consolidation chemotherapy’/exp OR ‘maintenance chemotherapy’/exp OR ‘medication therapy management’/exp OR ‘administration and dosage’/exp OR ‘injection site reaction’/exp) AND (‘educational technology’/exp OR ‘multimedia’/exp OR ‘teaching’/exp OR ‘video assisted technique’/exp OR ‘mass medium’/exp OR ‘health education’/exp OR ‘validation studies’ OR ‘validation studies as topic’ OR ‘validation study’/exp)*
*Web of Science*	*ALL=(“Nursing Care” OR “Patient Care Bundles” OR “Accident Prevention” ) AND ALL=(“Extravasation of Diagnostic and Therapeutic Materials” OR “Antineoplastic Agents” OR “Drug Therapy” OR “Chemotherapy, Adjuvant” OR “Induction Chemotherapy” OR “Consolidation Chemotherapy” OR “Maintenance Chemotherapy” OR “Medication Therapy Management” OR “Administration and Dosage” OR “Antineoplastic Combined Chemotherapy Protocols” OR “Injection Site Reaction”) AND ALL=(“Educational Technology” OR “Multimedia” OR “Teaching Materials” OR “Instructional Film and Video” OR “Communications Media” OR “Health Education” OR “Validation Study” OR “Validation Studies”)*
Scopus	*(( ALL ( “Nursing Care” OR “Patient Care Bundles” OR “Accident Prevention” ) ) AND ( ALL ( “Extravasation of Diagnostic and Therapeutic Materials” OR “Antineoplastic Agents” OR “Drug Therapy” OR “Chemotherapy, Adjuvant” OR “Induction Chemotherapy” OR “Consolidation Chemotherapy” OR “Maintenance Chemotherapy” OR “Medication Therapy Management” OR “Administration and Dosage” OR “Antineoplastic Combined Chemotherapy Protocols” OR “Injection Site Reaction” ) ) AND ( ALL ( “Educational Technology” OR “Multimedia” OR “Teaching Materials” OR “Instructional Film and Video” OR “Communications Media” OR “Health Education” OR “Validation Study” OR “Validation Studies” ) )*
CINAHL	*( MH “Nursing Care” OR “Nursing Care” OR “Patient Care Bundles” OR MH “Safety” OR “Accident Prevention” ) AND ( MH “Extravasation of Diagnostic and Therapeutic Materials” OR “Extravasation of Diagnostic and Therapeutic Materials” OR MH “Antineoplastic Agents” OR “Antineoplastic Agents” OR MH “Drug Therapy” OR “Drug Therapy” OR MH “Chemotherapy, Adjuvant” OR “Chemotherapy, Adjuvant” OR MH “Induction Chemotherapy” OR “Induction Chemotherapy” OR MH “Consolidation Chemotherapy” OR “Consolidation Chemotherapy” OR “Maintenance Chemotherapy” OR MH “Medication Management” OR “Medication Therapy Management” OR “Administration and Dosage” OR MH “Antineoplastic Agents, Combined” OR “Antineoplastic Combined Chemotherapy Protocols” OR MH “Injection Sites” OR “Injection Site Reaction” ) AND ( MH “Educational Technology” OR “Educational Technology” OR MH “Multimedia” OR “Multimedia” OR MH “Teaching Materials” OR “Teaching Materials” OR “Instructional Film and Video” OR MH “Communications Media” OR “Communications Media” OR MH “Health Education” OR “Health Education” OR MH “Validation Studies” OR “Validation Study”)*
SciELO	*((Nursing Care) OR (Patient Care Bundles) OR (Accident Prevention) ) AND ((Extravasation of Diagnostic and Therapeutic Materials) OR (Antineoplastic Agents) OR (Drug Therapy) OR (Chemotherapy, Adjuvant) OR (Induction Chemotherapy) OR (Consolidation Chemotherapy) OR (Maintenance Chemotherapy) OR (Medication Therapy Management) OR (Administration and Dosage) OR (Antineoplastic Combined Chemotherapy Protocols) OR (Injection Site Reaction)) AND ((Educational Technology) OR (Multimedia) OR (Teaching Materials) OR (Instructional Film and Video) OR (Communications Media) OR (Health Education) OR (Validation Study) OR (Validation Studies))*
LILACS	*((Nursing Care) OR (Patient Care Bundles) OR (Accident Prevention)) AND ((Extravasation of Diagnostic and Therapeutic Materials) OR (Antineoplastic Agents) OR (Drug Therapy) OR (Chemotherapy, Adjuvant) OR (Induction Chemotherapy) OR (Consolidation Chemotherapy) OR (Maintenance Chemotherapy) OR (Medication Therapy Management) OR (Administration and Dosage) OR (Antineoplastic Combined Chemotherapy Protocols) OR (Injection Site Reaction)) AND ((Educational Technology) OR (Multimedia) OR (Teaching Materials) OR (Instructional Film and Video) OR (Communications Media) OR (Health Education) OR (Validation Study) OR (Validation Studies))*
BDENF	*((Nursing Care) OR (Patient Care Bundles) OR (Accident Prevention)) AND ((Extravasation of Diagnostic and Therapeutic Materials) OR (Antineoplastic Agents) OR (Drug Therapy) OR (Chemotherapy, Adjuvant) OR (Induction Chemotherapy) OR (Consolidation Chemotherapy) OR (Maintenance Chemotherapy) OR (Medication Therapy Management) OR (Administration and Dosage) OR (Antineoplastic Combined Chemotherapy Protocols) OR (Injection Site Reaction)) AND ((Educational Technology) OR (Multimedia) OR (Teaching Materials) OR (Instructional Film and Video) OR (Communications Media) OR (Health Education) OR (Validation Study) OR (Validation Studies))*

A seleção incluiu estudos primários, independentemente do idioma ou ano de publicação. Por outro lado, não foram considerados estudos de caso, monografias, teses, dissertações, cartas ao editor, resumos, artigos de opinião, correspondências, resenhas ou capítulos de livros.

Após aplicar a estratégia de busca em cada base de dados, os resultados foram importados para o *software* de gerenciamento de referências *EndNote X7* (versão *Desktop*), onde ocorreu a eliminação dos artigos duplicados^(17)^. A seleção dos estudos foi conduzida utilizando a plataforma gratuita *Rayyan^®^*, desenvolvida pelo *Qatar Computing Research Institute*, visando identificar os que se enquadravam nos critérios de inclusão definidos previamente^(19)^.

A etapa preliminar da seleção consistiu na avaliação dos títulos e resumos dos trabalhos para verificar sua conformidade com os critérios de elegibilidade da RI. Posteriormente, os artigos aprovados passaram por uma leitura completa. Ambas as etapas foram conduzidas de forma independente e cega por dois revisores, fundamentados na questão da pesquisa e nos critérios de inclusão e exclusão estabelecidos. Em caso de discordância entre os avaliadores, um terceiro especialista na área foi acionado para dirimir as divergências.

Além das buscas realizadas nas bases de dados, os revisores também realizaram um levantamento manual nas referências bibliográficas dos estudos primários incluídos na RI.

### Caracterização e avaliação dos estudos

A obtenção dos dados foi realizada através de um instrumento readequado, que contemplou informações como referência e ano de publicação, objetivo do estudo, características metodológicas (incluindo o desenho do estudo conforme descrito pelos autores e o tamanho da amostra) e os principais achados relacionados às tecnologias educativas aplicadas na prevenção e no manejo do extravasamento de agentes antineoplásicos^(20)^.

Após esta etapa, a interpretação dos dados foi efetuada qualitativamente por meio da síntese descritiva das evidências provenientes dos estudos primários, conforme descrito pelos autores dos trabalhos incorporados na RI. Para o momento de avaliação crítica, foi escolhida a ferramenta do *Johns Hopkins Nursing Evidence-Based Practice* ^(21)^ para avaliar a consistência metodológica dos trabalhos escolhidos. Essa avaliação foi conduzida de forma independente por dois revisores, que aplicaram o instrumento específico para o desenho de estudo correspondente - seja quantitativo, qualitativo ou misto -, que utiliza respostas categorizadas como “sim”, “não” ou “não aplicável”. Na etapa anterior à avaliação crítica, os revisores definiram conjuntamente os critérios para julgamento, e, em situações de discordância, a decisão ficou a cargo de um terceiro revisor, que foi acionado para mediar e resolver os conflitos surgidos.

Os estudos selecionados foram classificados conforme a qualidade metodológica, divididos em três categorias: alta, boa e baixa qualidade. Estudos de alta qualidade apresentaram resultados sólidos e aplicáveis, amostras adequadas ao desenho da pesquisa, controle rigoroso, conclusões conclusivas e recomendações fundamentadas em uma revisão ampla da literatura, incluindo referências completas às evidências científicas. Aqueles considerados de boa qualidade mostraram resultados relativamente consistentes, amostras suficientes para o tipo de estudo, algum nível de controle, conclusões justificadas e recomendações baseadas em revisões abrangentes, ainda que com referências parciais às evidências. Por fim, estudos de baixa qualidade demonstraram evidências limitadas e resultados inconsistentes, amostras inadequadas ao desenho e conclusões não conclusivas^(21)^.

Reconhecendo a relevância de combinar a qualidade metodológica com o grau das evidências para apoiar decisões na prática clínica, os estudos selecionados foram organizados conforme os níveis de evidência estabelecidos pelo modelo hierárquico do *Johns Hopkins Nursing Evidence-Based Practice* ^(21)^.

## RESULTADOS

A consulta às bases de dados gerou um total de 2.272 registros recuperados, dos quais 259 foram identificados como duplicados e excluídos. Após a triagem inicial baseada na triagem dos títulos e resumos, de acordo com os critérios de inclusão e exclusão estabelecidos, seis artigos foram selecionados para leitura integral. Na etapa seguinte, a análise completa dos textos resultou na inclusão de três publicações nesta RI. O fluxo detalhado da identificação e seleção dos estudos é mostrado na Figura 1.

**Figura 1:** Representação gráfica do fluxo de identificação, triagem e inclusão dos estudos na revisão integrativa, 2025

O Quadro 2 apresenta um panorama descritivo dos estudos primários, com informações sobre autores, local e ano de publicação, tipo de estudo, amostra e objetivo, além de principais resultados e conclusões, tipo e classificação da tecnologia educativa, qualidade metodológica e nível de evidência, limitações de cada estudo, contexto de aplicação, e impacto prático.

**Quadro 2:** Caracterização dos estudos incluídos na revisão integrativa, 2025

Autores/local/ano	Tipo de estudo/amostra	Objetivo	Principais resultados/conclusões	Tipo/classificação da tecnologia educativa/Jhons Hopkins/NE	Limitações do estudo	Contexto de aplicação	Impacto prático
Melo *et al*., 2020, Brasil^(22)^	Estudo metodológico, desenvolvido em três fases: realização de revisão de escopo, construção de *bundle* e avaliação de conteúdo por especialistas. A amostra foi composta por 13 juízes na primeira etapa de Delphi e por nove juízes na segunda etapa de Delphi. Todos eram enfermeiros especialistas na área da oncologia.	Construir e avaliar o conteúdo de um *bundle* (conjunto de medidas) de prevenção e condutas diante do extravasamento de agentes antineoplásicos em pacientes oncológicos adultos.	O estudo validou um *bundle* de gerenciamento acerca do extravasamento de drogas vesicantes, mostrando que ele possui propriedades psicométricas aceitáveis e alta fidedignidade, com recomendações de alterações implementadas pelos juízes. A avaliação utilizou a técnica Delphi e alcançou alta concordância e significância. O *bundle* obteve Coeficiente de Validade de Conteúdo de 0,93, para prevenção, e 0,96, para condutas, ressaltando a importância da detecção precoce para prevenir danos maiores. A implementação do *bundle* pode melhorar a qualidade do cuidado oncológico, exigindo capacitação profissional e avaliações periódicas para garantir sua eficiência e aplicabilidade.	Bundle/leve-dura/alta qualidade/IIIc	Baixo número de respostas dos peritos.	Hospital de grande porte de Unidade de Alta Complexidade em Oncologia.	Reforçar a atenção dos enfermeiros quanto ao gerenciamento de condutas diante do extravasamento de drogas vesicantes em pacientes oncológicos.
Freitas, *et al*., 2022, Brasil^(23)^	Estudo descritivo de natureza qualitativa. A amostra foi composta por um grupo de enfermeiros especialistas.	Relatar a experiência na elaboração e implantação do protocolo de extravasamento e infiltração de antineoplásicos em acesso venenoso central.	A implementação de um protocolo para administração de agentes antineoplásicos reduziu significativamente complicações, padronizando procedimentos e aumentando a confiança dos profissionais de saúde. O protocolo facilitou a identificação prévia de sinais de infiltração e extravasamento, permitindo intervenções rápidas. A metodização melhorou a segurança e eficiência das práticas clínicas. Um fluxograma de cuidados foi instaurado em um ambulatório de oncologia em maio de 2021, acompanhado de capacitação para os funcionários.	Protocolo/leve-dura/baixa qualidade/IIIc	O estudo não apresenta, de forma explicita, suas limitações.	Ambulatório de oncologia do Hospital Estadual de Botucatu, vinculado ao Hospital das Clínicas da Faculdade de Medicina de Botucatu.	Aquisição de conhecimento e habilidades técnicas para o manejo da infiltração e extravasamento de agentes antineoplásicos em acesso venoso central.
Barbosa *et al*., 2024, Brasil^(9)^	Estudo de natureza metodológica, desenvolvido segundo o referencial teórico de Falkembach, que preconiza cinco etapas na construção de mídias educativas em vídeo: análise e planejamento, modelagem, implementação, avaliação e distribuição. A amostra foi composta por nove juízes com especialidade na área da oncologia e por cinco juízes especialistas na área de tecnologia e informação.	Construir e elaborar um vídeo educativo acerca da prevenção e manejo do extravasamento de agentes antineoplásicos direcionado a profissionais de enfermagem.	O vídeo educativo com duração de 18 minutos sobre gerenciamento do extravasamento de quimioterápicos alcançou um Índice de Validade de Conteúdo de 90,8%, com 94,2% entre as avaliações dos juízes de conteúdo e de técnica. Combinando narrativas e cenas simuladas, o vídeo aborda fatores de risco, ações preventivas e manejo adequado, com legendas para facilitar a compreensão. Bem recebido pelo público-alvo e juízes, foi considerado um método eficaz para capacitar enfermeiros e promover práticas baseadas em evidências na enfermagem.	Vídeo educativo/leve-dura/alta qualidade/IIIc	Tamanho do roteiro e *storyboard* em termos de conteúdo; duração final do vídeo; ausência de legendas em outros idiomas; e falta de recursos para o ensino de Libras a pessoas com deficiência auditiva.	Profissionais de enfermagem atuantes em serviços ambulatoriais de quimioterapia, especificamente na administração de agentes antineoplásicos.	A temática foi abordada de maneira acessível em plataformas digitais de acesso aberto, com potencial para melhorar o cuidado da pessoa com câncer, ultrapassando limitações geográficas.

Diante dos resultados, foi possível identificar que as tecnologias educativas apresentaram abordagens diversificadas na elaboração de recursos didáticos para o gerenciamento do extravasamento de quimioterápicos, tais como *bundle*, vídeo educativo e protocolo clínico. Essas tecnologias foram classificadas como leve-duras, por articularem saberes estruturados com elementos do trabalho em saúde.

O percurso metodológico para o processo de execução e o uso de uma fundamentação metodológica para o desenvolvimento das tecnologias educativas incluídos nesta revisão foram descritos em apenas dois estudos, demonstrando que as fases do processo metodológico foram corretamente implementadas e/ou detalhadas pelos autores, incluindo a especificação do método utilizado para a criação de recursos didáticos, assegurando o rigor em sua condução e demonstrando-se confiáveis.

Em síntese, as tecnologias, apesar de suas limitações, demonstraram que seus impactos na prática assistencial são estratégias inovadoras nos cuidados prestados em seu contexto de aplicação, além de construírem recursos didáticos que potencializam e facilitam o acesso à prática baseada em evidências direcionada aos profissionais de enfermagem. Ressalta-se que, em todos os estudos analisados, as ações de prevenção e manejo foram conduzidas por enfermeiros especializados em oncologia.

## DISCUSSÃO

O desenvolvimento de tecnologias educativas para orientar a atuação de profissionais diante do extravasamento de quimioterápicos persiste em uma lacuna importante para a prática clínica em enfermagem. O extravasamento de agentes antineoplásicos pode causar complicações severas, como necrose tecidual, dor intensa e perda funcional dos membros afetados^(1,2)^. Devido à complexidade do tratamento oncológico e ao risco significativo de danos aos pacientes, é fundamental que a equipe de enfermagem mantenham constante atualização sobre a adoção de práticas seguras para prevenir e tratar esses eventos^(9,22,23)^.

Nesse contexto, instituições responsáveis pela formação e assistência em saúde necessitam investir na elaboração de tecnologias educativas como recurso educativo, a fim de perpetuar ciclos de formação inovadores, através de uma abordagem construtivista e colaborativa, e viabilizar ferramentas educacionais que provoquem mudanças na implementação de estratégias eficazes para o cuidado de enfermagem^(24)^.

Os estudos incluídos^(9,22,23)^ reuniram evidências acerca das tecnologias educativas para suporte à prevenção e ao manejo de incidentes relacionados ao extravasamento, demonstrando que há poucos estudos voltados ao desenvolvimento e implementação de ferramentas educacionais específicas na temática, além de identificar que o percurso metodológico, que garante rigor à sua elaboração, foi empregado somente em dois estudos^(9,22)^, assegurando confiabilidade da tecnologia desenvolvida.

Em uma área como a oncologia, na qual novas drogas e protocolos são frequentemente introduzidos, a atualização constante do conhecimento entre profissionais de enfermagem é indispensável^(6-8)^. Sem acesso às ferramentas educativas eficazes, os profissionais podem não estar suficientemente preparados para identificar precocemente os sinais de extravasamento ou para implementar as intervenções necessárias de maneira eficiente, podendo resultar em respostas inadequadas ou tardias, aumentando o risco de complicações graves para os pacientes^(25)^.

As tecnologias têm o potencial de criar plataformas colaborativas que permitam a ampla disseminação de conhecimentos e experiências entre os profissionais de saúde. No entanto, sem estudos e iniciativas que promovam essas ferramentas, as oportunidades de aprendizado coletivo são limitadas, reduzindo a capacidade do sistema de saúde de responder de maneira proativa e eficaz aos desafios clínicos^(26)^. A adoção de ferramentas educativas tecnológicas na preparação dos profissionais de enfermagem tem se mostrado essencial para o manejo adequado do extravasamento de agentes antineoplásicos, uma emergência oncológica que pode causar danos graves^(9,22,23)^.

A síntese ora apresentada evidencia que a integração de ferramentas educacionais inovadoras, como protocolos^(23)^, vídeos^(9)^ e *bundles* ^(22)^, oferece suporte crucial para a formação contínua e prática baseada em evidências dos enfermeiros envolvidos no cuidado oncológico. A implementação de um protocolo para administração de antineoplásicos reduz significativamente complicações, padronizando procedimentos e aumentando a confiança dos profissionais de saúde^(23)^. O desenvolvimento de recursos educativos em formato audiovisual, por exemplo, é uma estratégia poderosa no contexto da enfermagem oncológica, e demonstra que vídeos educativos aumentam o entendimento dos profissionais sobre a prevenção e manejo do extravasamento, promovendo práticas seguras e baseadas em evidências^(9)^. Além disso, *bundles* educativos, que consistem em pacotes de práticas baseadas em evidências, têm se mostrado eficazes para a padronização dos cuidados^(22)^.

Embora os benefícios apontados sejam evidentes, cada tecnologia possui pontos fortes e limitações que devem ser avaliados no contexto da prática clínica. O vídeo educativo, embora acessível e de baixo custo, depende do engajamento ativo do profissional, não substituindo a supervisão prática ou a interação presencial. O protocolo clínico como pouco atrativo, do ponto de vista pedagógico, exige constate atualização para se manter alinhado às evidências. Já o *bundle* se configura como uma estratégia mais robusta, pois reúne intervenções integradas com potencial impacto nos desfechos clínicos; contudo, sua implementação demanda mais esforço institucional, incluindo treinamento, monitoramento contínuo e disponibilidade de recursos^(27-29)^. Assim, a escolha entre essas tecnologias e a combinação delas deve considerar não apenas sua eficácia isolada, mas também contextos organizacionais, a cultura de segurança vigente e os recursos disponíveis nos serviços de saúde.

Adicionalmente, a aplicação dessas tecnologias educativas deve ser alinhada às diretrizes internacionais de segurança do paciente oncológico. Organizações como a *Oncology Nursing Society* e o *Centers for Disease Control and Prevention* ressaltam a relevância de práticas padronizadas, comunicação eficaz e educação contínua da equipe de saúde para a prevenção de complicações^(30,31)^. Nesse contexto, protocolos e *bundles* oferecem suporte para padronização e implementação de medidas baseadas em evidências, enquanto vídeos educativos contribuem para o entendimento e adesão às práticas recomendadas^(9,22,23)^. Dessa forma, a integração dessas tecnologias não apenas fortalece a formação contínua dos enfermeiros, mas também promove conformidade com recomendações internacionais, reforçando práticas seguras e consistentes no cuidado oncológico^(30,31)^.

Mesmo com o potencial demonstrado, falta de tecnologias educativas amplamente disponíveis e validadas no contexto do extravasamento de agentes antineoplásicos representa, portanto, uma lacuna crítica na oncologia, com impactos diretos no aprimoramento do cuidado e proteção do paciente. A ausência dessas ferramentas pode resultar em um manejo ineficaz e aumentar a carga de trabalho dos profissionais de saúde. A formação inadequada pode levar à aplicação de técnicas desatualizadas e inconsistentes, além de dificultar a implementação de práticas baseadas em evidências^(6-8,32)^.

As tecnologias educativas consistem em um saber ampliado pela intervenção humana, não apenas na concepção e implementação de ferramentas. Constitui um conjunto ordenado de conhecimentos com propósito pedagógico, embasado em evidências científica, que possibilitam a organização, a efetivação, o domínio e a avaliação contínua do processo educativo^(10,33)^. Nessa perspectiva, as especificidades dos recursos didáticos evidenciam o predomínio de tecnologias do tipo leve-duras, que contribuíram para a adoção de medidas de prevenção e manejo do extravasamento direcionadas aos profissionais de enfermagem entre os estudos incluídos nesta RI.

É notório que as tecnologias contribuem com o cenário da enfermagem, sendo aplicadas com finalidade assistencial e educativa, reconhecidas como instrumento educacional que envolve múltiplos aspectos, incluindo texto, som e imagens, em único material de avanço do conhecimento^(34,35)^. Em adição, sua aplicação incrementa as competências dos profissionais na prática assistencial e oportuniza o saber de habilidades essenciais para o cuidado, uma vez que favorece o aprendizado e melhora tanto o desempenho quanto a satisfação da equipe^(36,37)^.

A maioria dos estudos apresentou nível de evidência IIIc, com dois deles demonstrando alta qualidade na avaliação metodológica, além da utilização de uma fundamentação técnica com o objetivo de guiar as fases de elaboração do recurso educativo. Por fim, é notável que o desenvolvimento e o uso de tecnologias educativas estão cada vez mais evidentes na formação dos profissionais de enfermagem, e alinhados a esse fenômeno, os enfermeiros brasileiros têm apresentado posição de destaque na concepção desse tipo de material.

Investir em tecnologias educativas e em pesquisas nesta área é essencial para melhorar a qualidade do cuidado e assegurar que os profissionais estejam aptos para superar os desafios associados ao gerenciamento do extravasamento de quimioterápicos. É urgente abordar essa lacuna, promovendo um ambiente onde a educação permanente e o acesso a informações baseadas em evidências sejam garantidos para todos os profissionais envolvidos no cuidado oncológico.

### Limitações do estudo

Uma limitação deste estudo foi a baixa quantidade de publicações identificadas sobre tecnologias educativas relacionadas à temática investigada, com apenas três estudos encontrados. Esse resultado pode comprometer a solidez das conclusões, pois a escassez de registros sugere uma possível lacuna na produção científica da área. Além disso, a quantidade reduzida de estudos pode refletir uma menor preocupação ou envolvimento dos profissionais de enfermagem no desenvolvimento de novas tecnologias, evidenciando a necessidade de maior investimento na criação e disseminação de estratégias educativas. Outra limitação é a restrição geográfica dos estudos, uma vez que todos foram desenvolvidos no Brasil. Essa limitação impede uma visão mais ampla sobre a temática em diferentes contextos e realidades, dificultando a comparação com experiências internacionais. Esse cenário reforça a importância de novas pesquisas em outras regiões e países, a fim de expandir o conhecimento sobre o desenvolvimento e a aplicação de tecnologias educativas na área investigada.

### Contribuições para a área da enfermagem

Esta RI traz contribuições significativas para o avanço do conhecimento e para as práticas dos profissionais de enfermagem. Devido à gravidade das complicações associadas ao extravasamento de agentes antineoplásicos, é essencial desenvolver materiais educativos que possam preencher essas lacunas. Estudos bem fundamentados podem oferecer diretrizes claras e práticas baseadas em evidências, contribuindo para a padronização dos cuidados e minimização dos riscos para os pacientes. Além disso, ao abordar as limitações atuais, tais estudos incentivam a realização de pesquisas futuras mais abrangentes e diversificadas, promovendo uma melhoria contínua na segurança e eficácia do tratamento oncológico.

## CONCLUSÕES

As evidências consolidadas nesta revisão indicam que as tecnologias educativas para o gerenciamento do extravasamento de quimioterápicos ainda são pouco exploradas na literatura científica, o que pode refletir uma lacuna na qualificação dos profissionais de enfermagem e na adoção de estratégias sistematizadas para a segurança do paciente oncológico.

No entanto, ao considerar as evidências sintetizadas, conclui-se que os recursos didáticos contribuem para o aprimoramento do cuidado oncológico na prática clínica, configurando-se como métodos eficazes para capacitar enfermeiros, promover práticas baseadas em evidências na enfermagem e reduzir complicações associadas ao extravasamento. Essas estratégias não apenas reforçam a segurança do paciente, mas também aprimoram a qualidade da assistência prestada.

## Data Availability

Os dados de pesquisa estão disponíveis no corpo do artigo.
